# Anaphylatoxin Complement 5a in Pfizer BNT162b2-Induced Immediate-Type Vaccine Hypersensitivity Reactions

**DOI:** 10.3390/vaccines11061020

**Published:** 2023-05-23

**Authors:** Xin Rong Lim, Grace Yin Lai Chan, Justina Wei Lynn Tan, Carol Yee Leng Ng, Choon Guan Chua, Guat Bee Tan, Stephrene Seok Wei Chan, Kiat Hoe Ong, Ying Zhi Tan, Sarah Hui Zhen Tan, Claire Min Li Teo, Samuel Shang Ming Lee, Bernard Yu Hor Thong, Bernard Pui Lam Leung

**Affiliations:** 1 Department of Rheumatology, Allergy and Immunology, Tan Tock Seng Hospital, Singapore 308433, Singaporecarol_ng@ttsh.com.sg (C.Y.L.N.); bernard_thong@ttsh.com.sg (B.Y.H.T.);; 2 Department of Haematology, Tan Tock Seng Hospital, Singapore 308433, Singapore; 3 Health and Social Sciences, Singapore Institute of Technology, Singapore 138683, Singapore

**Keywords:** COVID-19 vaccines, anaphylaxis, hypersensitivity reactions, pseudo-allergic reactions, anaphylatoxins, complement 5a

## Abstract

The underlying immunological mechanisms of immediate-type hypersensitivity reactions (HSR) to COVID-19 vaccines are poorly understood. We investigate the mechanisms of immediate-type hypersensitivity reactions to the Pfizer BNT162b2 vaccine and the response of antibodies to the polyethylene glycol (PEG)ylated lipid nanoparticle after two doses of vaccination. Sixty-seven participants, median age 35 and 77.3% females who tolerated two doses of the BNT162b2 vaccine (non-reactors), were subjected to various blood-sampling time points. A separate group of vaccine reactors (10 anaphylaxis and 37 anonymised tryptase samples) were recruited for blood sampling. Immunoglobulin (Ig)G, IgM and IgE antibodies to the BNT162b2 vaccine, biomarkers associated with allergic reaction, including tryptase for anaphylaxis, complement 5a(C5a), intercellular adhesion molecule 1 (ICAM-1) for endothelial activation and Interleukin (IL)-4, IL-10, IL-33, tumour necrosis factor (TNF) and monocyte chemoattractant protein (MCP-1), were measured. Basophil activation test (BAT) was performed in BNT162b2-induced anaphylaxis patients by flow cytometry. The majority of patients with immediate-type BNT162b2 vaccine HSR demonstrated raised C5a and Th2-related cytokines but normal tryptase levels during the acute reaction, together with significantly higher levels of IgM antibodies to the BNT162b2 vaccine (IgM 67.2 (median) vs. 23.9 AU/mL, *p* < 0.001) and ICAM-1 when compared to non-reactor controls. No detectable IgE antibodies to the BNT162b2 vaccine were found in these patients. The basophil activation tests by flow cytometry to the Pfizer vaccine, 1,2-dimyristoyl-rac-glycero-3-methoxypolyethylene glycol (DMG-PEG) and PEG-2000 were negative in four anaphylaxis patients. Acute hypersensitivity reactions post BNT162b2 vaccination suggest pseudo-allergic reactions via the activation of anaphylatoxins C5a and are independent of IgE-mechanisms. Vaccine reactors have significantly higher levels of anti-BNT162b2 IgM although its precise role remains unclear.

## 1. Introduction

As of 14 August 2022, the coronavirus disease 2019 (COVID-19) global pandemic has infected over 587 million people worldwide and caused 6.4 million fatalities [1]. Singapore has reported a total of 1.79 million cases and 1539 deaths by 7 August 2022 thus far [2]. There has been unprecedented socioeconomic disruption globally and fears about COVID-19′s uncontrollable spread and the deadliness of the disease.

To curb this pandemic and restore normalcy globally, vaccines against SARS-CoV-2 were developed and made available for emergency use in less than 12 months. The United States (US) Food and Drug Administration (FDA) granted emergency use authorization for the Pfizer-BioNTech vaccine on 11 December 2020. On 6 January 2021, the US Centers for Disease Control (CDC) announced that there had been 21 cases of anaphylaxis out of 1,893,360 first doses of the Pfizer-BioNTech vaccine administered between 14 and 23 December, a reaction rate of 11.1 cases per million doses. Most of the reactions (71%) occurred within 15 min of vaccination [3]. In comparison, anaphylaxis with influenza vaccines occurs at a rate of 1.31 cases per million doses [4].

Singapore’s Health Sciences Authority (HSA) granted interim authorisation for the Pfizer BNT162b2 vaccine under the Pandemic Special Access Route (PSAR) on 14 December 2020 and the Moderna mRNA-1273 vaccine on 3 February 2021 [5,6]. The first safety update by the HSA reported 20 cases of anaphylaxis with Pfizer and Moderna messenger RNA (mRNA) vaccines, a reaction rate of 1.4 per 100,000 doses administered [7]. The 28 February 2022 HSA safety update reported the incidence rate of anaphylaxis with mRNA vaccines is low, at 0.67 per 100,000 doses administered [8]. 

The precise mechanisms resulting in severe hypersensitivity reactions (HSR) to mRNA COVID-19 vaccines have not been determined although these appear to be heterogeneous [9,10,11]. The mechanisms have been broadly classified as allergic or pseudoallergic. The Pfizer-BioNTech COVID-19 vaccine (BNT162b2) is a messenger RNA (mRNA) vaccine that uses lipid nanoparticles to facilitate the transport of mRNA into cells. This vaccine contains several excipients and lipids and polyethylene glycol (PEG)-2000 is one of the excipients with recognised allergenic potential. The clinical and research communities have postulated that such reactions could be due to IgE-mediated mechanisms via anti-PEG IgE or other mechanisms via anti-PEG IgM or anti-PEG IgG antibodies [12,13,14]. We aim to investigate the mechanisms of immediate-type reactions to the Pfizer BNT162b2 vaccine and the response of antibodies to the polyethylene glycol (PEG)ylated lipid nanoparticle after two doses of Pfizer BNT162b2 vaccination.

## 2. Materials and Methods

This observational study was designed to study various biomarkers and the level of antibodies to PEGylated lipid nanoparticles in vaccine reactors and vaccine non-reactors. 

### 2.1. Participant Selection

Vaccine non-reactors comprise adult participants (all 21 years old and above) who were patients recruited from our institution’s Allergy clinic attending the clinic for a challenge dose due to non-COVID-19 vaccine allergy [15] and staff in our hospital receiving vaccinations from the occupational health clinic. The following patients/conditions were excluded from the study: pregnant women, active malignancy on treatment, systemic rheumatic diseases and those with prior SARS-CoV-2 infection. Participants completed a questionnaire on their atopy history and a survey on post-vaccination reactions. 

Vaccine reactors comprise 2 groups: patients who developed anaphylaxis with the first or second dose of the BNT162b2 vaccine and anonymized reactors. Ten patients with anaphylaxis after the first or second dose of the BNT162b2 vaccine were recruited from our institution’s Allergy clinic or inpatient wards. These patients were assessed by an allergist and diagnosed with anaphylaxis to the BNT162b2 vaccine. The assessment and evaluation of the mRNA vaccine allergy included a comprehensive history-taking of the details of the reaction, reviewing the patient signs during the reaction, laboratory results, treatment given, outcome as well as recovery. Anaphylaxis was defined using the Brighton Collaboration Anaphylaxis Working Group’s case definition [16]. Our institution’s Clinical Immunology Laboratory (CIL) receives and processes nationwide samples for tryptase testing for acute hypersensitivity reactions due to the mRNA COVID-19 vaccine. Anonymized reactors from other institutions were selected based on the clinical history and diagnosis of anaphylaxis in reaction to the Pfizer BNT162b2 vaccine made by clinicians on the laboratory request form. Tryptase samples and leftover sera from 37 anonymized reactors were included in the study.

The study was approved by the institutional review board (National Healthcare Group, Domain Specific Review Board reference number: 2021/00174) and written informed consent was obtained from participants. A subsequent approval for waiver of informed consent for use of anonymised leftover sera from tryptase samples was obtained from our institutional review board (DSRB 2021/00878).

### 2.2. Sample Collection

Vaccine non-reactors were divided into 2 groups with different pre- and post-vaccination blood collection as detailed below:

(a) Group A: Blood sampling pre-vaccination, 1 h post first dose BNT162b2 vaccine, 8 weeks post second dose BNT16b2 vaccine;
(b) Group B: Blood sampling pre-vaccination, 1 h post second dose BNT162b2 vaccine, 8 weeks post second dose BNT16b2 vaccine.

We had initially planned to obtain blood sampling within 2 h of the reaction for patients who presented with anaphylaxis. However, there were difficulties achieving this as often the focus during the acute reaction will be on resuscitation of patient and management of the acute reaction. Blood sampling was performed within 6 h (Range: 3–6 h) in 6 anaphylaxis patients and within 6 to 72 h in the remaining 4 patients. 

As for the 37 anonymised reactors, leftover tryptase sera from these 37 individuals’ samples were used. The timing of these blood samples with respect to the reaction is unknown. The majority of these patients have serial tryptase samples sent and the first tryptase sample was used. 

### 2.3. Immunological Methods

Biomarkers that were assayed included antibodies to BNT162b2, complement 5a(C5a), intercellular adhesion molecule 1 (ICAM-1) for endothelial activation and cytokine analysis including Interleukin (IL)-4, IL-10, IL-33, tumour necrosis factor (TNF) and monocyte chemoattractant protein (MCP-1). 

Pfizer BNT162b2 was employed as capture antigen by immobilising onto enzyme-linked immunosorbent assay (ELISA) plate overnight in 0.1 M of NaH_2_CO_3_, (pH8.6) at 1:25 dilution. Anti-Pfizer BNT162b2 IgE antibody titres of individual sera were detected with biotin-conjugated anti-human IgE (BD Biosciences, San Diego, USA) as previously described [17]; human anti-PEG IgE was employed as standard (0.2–1000 ng/mL) based on the supplier’s recommendation (Hu 6.3 IgE, Academia Sinica, Taipei, Taiwan). Anti-Pfizer BNT162b2 IgG (0.2–1000 ng/mL, Hu 6.3 IgG, Academia Sinica) and anti-Pfizer BNT162b2 IgM were measured in a similar manner and expressed as arbitrary unit (AU/mL). The lowest reliable detection limit was 2.5 standard deviations (SD) from blank. Sera from 24 healthy unvaccinated subjects collected prior to COVID-19 pandemic were used to determine the baseline levels, with anti-BNT162b2 IgG 105 ng/mL (65.0–217.8, median and 95% confidence intervals (CI)) and anti-BNT162b2 IgM 17.9 AU/mL (11.7–28.4). Human complement C5a, IL-33, ICAM-1 (R&D Systems, Abingdon, UK), IL-4 and MCP-1 (BD Biosciences) were assayed by ELISA according to the manufacturers’ instructions, with detection limit as follow: IgG and IgE (<0.2 ng/mL), IL-10 and TNF (<0.1 pg/mL); IL-4 and IL-33 (<1 pg/mL), C5a and ICAM-1 (<0.12 ng/mL). The presence of SARS-CoV-2 neutralisation antibody post Pfizer BNT162b2 vaccination was assayed by cPass SARS-CoV-2 neutralisation ELISA (GenScript Biotech, Singapore), which is a blocking ELISA measuring the total IgM and IgG response against the receptor binding domain (RBD) protein from the SARS-CoV-2 spike (S) protein. A positive value of 30% or greater is defined as the presence of SARS-CoV-2 neutralisation antibody based on 50% reduction to plaque reduction neutralization (PRNT_50_) test according to WHO guidelines.

Basophil activation test (BAT) was performed by flow cytometry (Flow CAST, Bühlmann Laboratories AG, Schönenbuch, Switzerland) according to manufacturer’s instructions. Freshly collected heparinized whole blood was incubated in reaction buffer with Pfizer BNT162b2 vaccine at 1/100 (1%) and 1/1000 (0.1%) dilutions, PEG-2000, 1,2-dimyristoyl-rac-glycero-3-methoxypolyethylene glycol-2000 (DMG PEG-2000, both at 1%) or positive control of monoclonal antibody against human IgE Fc receptor I (anti-FceRI mAb) with final concentrations similar to previous studies [18,19]. Basophil degranulation was determined through detection of surface CCR3 (PE), CD63 (FITC) and CD203c (PE-DY647) expression. As BAT was not available locally during the initial phase of Pfizer BNT162b2 vaccination, BAT was performed 8 to 12 months subsequently from the reaction in 5 patients with anaphylaxis. 

### 2.4. Statistical Analysis

Patient characteristics were summarised using descriptive analyses. Continuous variables were expressed as medians (interquartile range, IQR); one-way ANOVA was used for parametric data and Mann–Whitney U test for non-parametric variables. Data analyses were conducted using Prism 8 (GraphPad Software, San Diego, CA, USA), with *p*-value < 0.05 considered statistically significant.

## 3. Results

**Figure 1:**
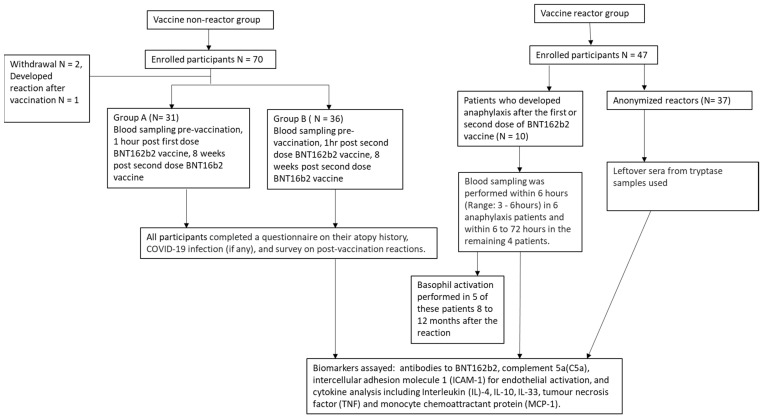
Flow diagram of study participants.

Between 1 June 2020 and 30 September 2020, we recruited 70 participants in the non-reactors group. Two participants withdrew their consent and one participant developed periorbital angioedema after the second BNT162b2 vaccine and was excluded from the final analysis. In total, 67 participants were included in the final analysis. There were 31 participants in Group A and 36 participants in Group B (Figure 1). Between 1 January 2020 and 30 May 2021, 10 patients with anaphylaxis in response to the first or second dose of BNT162b2 vaccine and 37 anonymised reactors were identified. All 67 vaccine non-reactors and the 10 patients with anaphylaxis did not have COVID-19 infection prior to vaccination. 

The demographics and atopy history of the 10 anaphylaxis patients and 67 vaccine non-reactors are shown in Table 1. 

Of the 10 anaphylaxis patients, 7 developed reactions after the first dose of the BNT162b2 vaccine (Table 2). Two of the patients who developed immediate reactions after the second dose of BNT162b2 experienced mild reactions after the first dose. Further details of the laboratory findings of these 10 patients are available in Supplementary Table S1. Six patients with anaphylaxis had tryptase levels performed within 6 h of an acute reaction with all demonstrating normal tryptase reference levels (<11.4 ng/L). Baseline tryptase was followed up in these six patients and the acute tryptase samples did not show a significant rise (>1.2x baseline tryptase + 2 ng/L). Similarly, all 37 anonymised tryptase samples also demonstrated tryptase within normal reference levels. 

The antibody levels against immobilised Pfizer BNT162b2 as antigens from non-reactors 1 h post the first or second dose BNT162b2 vaccine were compared with reactors. There was no significant difference in the antibody levels in non-reactors after subcategorizing them into post first or second vaccination. The reactors comprised 10 patients with anaphylaxis and 37 anonymised tryptase samples. The vaccine reactors demonstrated significantly higher median levels of anti-BNT162b2 IgM levels compared to the non-reactors (Table 3(a)). No detectable IgE antibodies to BNT162b2 were found in the samples, while the anti-BNT162b2 IgG levels were found to be similar between both groups. The reactors also demonstrated significantly higher median levels of complement C5a (39.4 vs. 160 ng/mL, *p* < 0.001) and intercellular adhesion molecule ICAM-1 (46.03 vs. 136 ng/mL, *p* < 0.001) as an endothelial activation marker (Table 3(a)). Similarly, the six anaphylaxis patients with samples taken within 6 h of the hypersensitivity reaction demonstrated higher median (IQR) levels of anti-BNT162b2 IgG and IgM levels, C5a and ICAM-1 compared to the non-reactors (Table 3(b)). 

Among the anaphylaxis patients, 83% (5/6) with blood collection within 6 h of the onset of the reaction and 86% (32/37) of the anonymised tryptase samples demonstrated elevated levels of anaphylatoxin C5a. As the anaphylactic patients and the anonymised reactors had serial tryptase samples sent off, the subsequent samples performed >24 h after the reaction showed that the C5a levels reduced with time. We decided to separate these groups of vaccine reactors according to the C5a levels; we employed an empirical data-driven approach on C5a levels from the 67 vaccine non-reactors, where 95% of the data fell within mean + 2.5 standard derivations, with the final cut-off of 76.6 ng/mL denoting elevated C5a. By separating the vaccine reactors based on complement activation, the reactors with high C5a demonstrated significantly higher levels of allergic Th2 cytokines IL-4, IL-33 and MCP-1 compared to the reactors with low C5a (Table 4), demonstrating two independent subsets in the absence of IgE antibodies.

We next performed BAT by flow cytometry to study the effects of Pfizer-BNT162b2 on basophil degranulation on five anaphylaxis patients. None of the anaphylaxis patients demonstrated a positive BAT to the BNT162b2 vaccine (0.1%), DMG PEG-2000 or PEG-2000 (Table 5). Patient #1 BAT was excluded as the positive control (anti-IgE Fc receptor) failed to achieve the required activation of >10% basophils.

**Figure 2:**
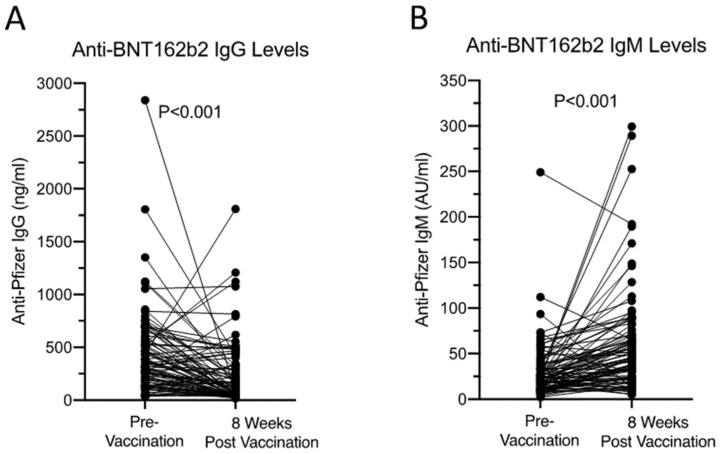
Anti-BNT162b2 IgG (**A**) and IgM (**B**) levels pre- and 8 weeks post-vaccination from 67 non-reactors. All received two doses of Pfizer BNT162b2 vaccine 3 weeks apart. Samples were taken pre-vaccination and 8 weeks after the second vaccination. Mann–Whitney U test was used to assess the statistical difference between the two groups.

The anti-BNT162b2 IgG and IgM levels at pre- and 8 weeks post-vaccination from the 67 non-reactors were studied next. We observed an overall reduction in anti-BNT162b2 IgG levels 8 weeks after the second vaccination from 321.8 (117.9–571.3, median and IQR) to 83.1 (42.5–312.7) ng/mL, *p* < 0.001. Anti-BNT162b2 IgM increased 8 weeks after the second vaccination from 21.9 (11.4–34.4) to 45.8 (23.6–71.0) AU/mL (*p* < 0.001, Figure 2). Finally, IL-10 is known to promote humoral immune responses inducing immunoglobulin (Ig) production [20]. The serum IL-10 levels in these 67 samples were assayed and all were found to be within the normal healthy cut-off (2 pg/mL), suggesting changes in anti-BNT162b2 IgM and IgG are independent of the IL-10 pathway.

## 4. Discussion

We were unable to detect any IgE antibodies to the BNT162b2 vaccine nor elevated tryptase levels in our anaphylaxis patients and anonymised tryptase samples. Instead, the majority of these vaccine reactors demonstrated raised levels of C5a, suggesting complement activation and the subsequent activation of cytokines IL-4, IL-10, IL-33, MCP-1. These cytokines have been reported to be elevated in anaphylaxis and correlate with the severity of the anaphylaxis [21,22]. 

We previously reported three patients with anaphylaxis post Pfizer BNT162b2 vaccine with similar laboratory findings of normal tryptase levels, a lack of IgE antibodies to the BNT162b2 vaccine and high C3a and enhanced Th2 cytokine profile levels [17]. Similar to our observation, Jiang et al., 2021, reported a case of anaphylaxis post BNT162b2 vaccine with normal tryptase during the acute reaction, a negative skin prick test to the vaccine, DMG-PEG and polysorbate 80 and low levels of PEG IgE antibodies [23]. Warren et al. found that the majority of the subjects with suspected allergic reactions to the mRNA vaccine had negative skin prick testing to PEG, polysorbate and the mRNA vaccine [18]. No PEG IgE was detected, but PEG IgG was found instead. 91% and 100% of their participants had a positive BAT to PEG and the mRNA vaccine, respectively. However, we were unable to demonstrate any positive BAT in our anaphylaxis patients. Emerging evidence has shown that PEG conjugated with lipid nanoparticles rather than PEG alone induces positive basophil activation in patients with a PEG allergy, suggesting that the structure or form of PEG plays a role in potentially triggering allergic reactions [19]. 

Studies have shown that the majority of patients who experienced convincing immediate HSR to the first dose of the mRNA vaccine were able to tolerate a second dose without severe complications, some with antihistamine or corticosteroid pre-medication [24,25,26]. These findings have led to expert opinion that these mRNA vaccine HSR are not due to classical IgE-mediated reactions, as IgE-mediated reactions typically cause similar or worse reactions upon re-exposure to the same drug. 

Polyethylene glycol has not been previously used as an excipient in vaccines, but PEG of varying molecular weights is commonly found in household products [27]. Yang et al. reported the presence of anti-PEG IgG and IgM in 89% of people with prior exposure to PEG-containing products [28]. Notably, PEG-related allergies may be more common in the female population based on PEG-containing household products and cosmetics. Given the widespread prevalence of PEG-containing ingredients, the exposure to these products may lead to sensitization and subsequent allergic events occurring following first-time exposure to parenteral PEG products [29,30].

Our study had more female participants in both groups. Vaccine non-reactors comprised patients attending the Allergy clinic for a challenge dose of BNT162b2 vaccine due to non-COVID-19 vaccine allergy and hospital staff attending the occupational health clinic for BNT162b2 vaccination. Possible reasons for the higher number of female participants in the non-reactor group include the more proactive health-seeking behaviour of females compared to males and the predominance of females in the nursing and allied health professions. Similar to other studies, our study also demonstrates female predominance in mRNA COVID-19 vaccine anaphylaxis cases [31].

The postulated non-IgE-mediated mechanisms of mRNA COVID-19 hypersensitivity reactions include complement activation-related pseudoallergy (CARPA) or direct activation by PEGylated liposome. Complement activation-related pseudoallergy occurs when exposure to PEGylated liposomes of the vaccine causes pre-existing anti-PEG IgG/IgM to trigger the generation of anaphylatoxins C3a and C5a. These anaphylatoxins activate allergic effector cells that kickstart the inflammatory mechanism. Additionally, these PEGylated liposomes can also bind to the allergic effector cells to trigger inflammation [12]. While IgE-independent pseudoanaphylaxis has been clearly demonstrated in animal models with characteristic blood cell changes including thrombocytopenia, leukocytosis and leukopenia, it is still not clearly demonstrable in human pathology [32,33]. Clinical reports of haematological changes including leukopenia in response to drugs may be a cytotoxic effect of the drug rather than due to the immunological changes from the reaction to the drugs [34]. None of our patients demonstrated such haematological changes apart from mild leukopenia. It still remains unclear whether anti-PEG IgM and IgG result in non-IgE-mediated reactions or complement activation-related pseudoallergy (CAPRA) in patients with mRNA vaccine allergies.

While our data show that vaccine reactors have higher levels of anti-BNT162b2 IgM, we observe that some participants despite having high levels of anti-BNT162b2 IgM do not react to the vaccine. Conversely, there are vaccine reactors with raised C5a levels during the acute reaction but their anti-BNT162b2 IgM levels are not high. Anti-BNT162b2 IgG and IgM levels prior to vaccination were lacking in the anaphylaxis patients and the antibody levels were measured after the reaction. It is possible that these antibodies have bound to certain targets and are therefore not detectable in the bloodstream. By separating the vaccine reactors into two groups (high and low C5a), we see there is no difference in the levels of anti-BNT162b2 IgG and IgM between the two groups. The presence of these IgG and IgM antibodies alone did not appear to be able to trigger reactions via the generation of anaphylatoxin C5a. 

The effect of the mRNA vaccine in inducing PEGylated antibodies is unclear. Yi Ju et al. reported the rise in PEG-specific antibodies following BNT162b2 and mRNA-1273 vaccination, with an increase in both anti-PEG IgG and IgM [35]. In contrast, Guerrini, G. et al. reported a significant increase in anti-PEG IgM after the first and third mRNA vaccine but no significant increase in anti-PEG IgG levels after vaccination [36]. Our novel ELISA assay to detect antibodies to the Pfizer BNT162b2 vaccine involves immobilising the Pfizer BNT162b2 vaccine on ELISA plates to allow the binding of antibodies in serum samples against all the potential immunogenic epitopes of the vaccine. We postulate that the high prevalence of pre-existing IgG and IgM antibodies in response to the Pfizer BNT162b2 vaccine in the vaccine non-reactors prior to vaccination were antibodies against the PEGylated component of the vaccine. To investigate whether these antibodies to the Pfizer BNT162b2 vaccine affect vaccine efficacy, SARS-CoV-2 Neutralization Antibody responses were examined via blocking ELISA measuring the total IgM and IgG response against the receptor binding domain (RBD) protein from the SARS-CoV-2 spike (S) protein in the vaccine non-reactors 8 weeks after the second vaccination. All 67 vaccine non -reactors except for 1 individual developed positive responses to the SARS-CoV-2 Neutralization Antibody (Positive = >30%) 8 weeks after the second vaccination. The particular individual who did not achieve positive responses to the SARS-CoV-2 Neutralization Antibody did not have high levels of anti-Pfizer IgG and IgM antibodies. Our study shows that anti-BNT162b2 IgG levels decrease 8 weeks after vaccination while anti-BNT162b2 IgM levels increase. It appears that there are certain immunoregulatory mechanisms that prevent the further production of anti-BNT16b2 IgG. This could explain the tolerability of the subsequent second dose vaccine in those who are first-dose allergic. Overall, the clinical relevance of these antibodies induced by the vaccine and future interactions with other PEGylated drugs (e.g., chemotherapeutic agents) and subsequent doses of the mRNA vaccine (e.g., enhanced primary series or boosters in individuals at high risk of severe COVID-19 infection) remains unclear. 

The weakness of our study is the lack of clinical details of the anonymised reactor tryptase samples as they were received from other institutions. The blood sampling performed in the anaphylaxis group was not homogenous across all patients as it was difficult to obtain blood samples during an acute reaction as the focus was on management of the patient’s allergic reactions. Four of our anaphylaxis patients’ blood draws were delayed up to 72 h and the normal serum tryptase levels in these individuals cannot exclude the possibility of either IgE- or non-IgE-mediated mast cell degranulation. Skin testing for PEG/polysorbate and the Pfizer BNT162b2 vaccine was not performed as the efforts of the allergist were channelled to evaluating patients with reported non-COVID-19 vaccine and/or PEG allergies prior to mRNA vaccination expediently before the next COVID-19 surge. Moreover, several studies have emerged on the limited role of excipient skin testing in those with suspected reactions to the mRNA COVID-19 vaccine [26]. In addition, our study is underpowered for further sub-group analyses in Table 3(b) and Table 4, and may be subjected to Type II error. Finally, there were also restricted stocks of vaccines procured and complex logistics of obtaining vaccines from leftover vials following reconstitution during the peak of the National Vaccination Program [15].

## 5. Conclusions

We demonstrated elevated levels of C5a and Th2 allergic cytokines in individuals with immediate hypersensitivity to BNT162b2 vaccines but the exact immunological mechanisms triggering anaphylatoxin and cytokine production were unclear. Emerging evidence and studies suggest that these vaccine hypersensitivity reactions are possibly non-IgE-mediated. The role of anti-PEG IgG and IgM as a cause of inducing these reactions remains to be explored.

## Figures and Tables

**Table 1:** Demographics, atopy history and vaccine reactions in vaccine non-reactors and patients with anaphylaxis in response to BNT162b2 vaccine.

Demographics	Vaccine Non-Reactors *N* = 67	Anaphylaxis Patients *N* = 10
Age, mean (SD)	35 (17.5)	43.4 (14.5)
Sex (female, %)	75(77.3%)	8 (80%)
Race (%)		
Chinese	71 (73.2%)	8 (80%)
Malay	14 (14.4%)	0 (0%)
Indian	4 (4.12%)	2 (20%)
Others	8 (8.25%)	0 (0%)
Allergic and atopic conditions		
Allergic rhinitis	15 (15.5%)	6 (60%)
Asthma	10 (10.3%)	5 (50%)
Chronic spontaneous urticaria	10 (10.3%)	2 (20%)
Food allergy	28 (28.9%)	3 (30%)
Eczema	12 (12.4%)	1 (10%)
Drug allergy/hypersensitivity	13 (23.4%)	4 (40%)
Vaccine reactions		
Cutaneous	N.A.	9 (90%)
Upper airway	N.A.	8 (80%)
Lower airway	N.A.	10 (100%)
Cardiovascular	N.A.	2 (20%)
Gastrointestinal	N.A.	3(30%)
Brighton Level 1	N.A.	6 (60%)
Brighton Level 2	N.A.	4 (40%)

Legend: Cutaneous: pruritus, rash (urticarial and non-urticarial), angioedema, flushing; Upper airway: Throat swelling, hoarse voice, globus; Lower airway: wheezing, cough, breathlessness; Cardiovascular: Tachycardia and hypotension; Gastrointestinal: nausea, vomiting, abdominal pain, diarrhoea.

**Table 2:** Clinical presentation of 10 patients with anaphylaxis in reaction to Pfizer BNT162b2 vaccine.

S/N	Age	Gender	Ethnicity	Reaction Onset	Vaccine Dose	Atopy History	Signs and Symptoms	Brighton Level	Treatment
1	45	M	Indian	30 min	Second	Asthma, urticaria to etoricoxib	Flushing, periorbital edema, globus sensation, wheezing * Developed periorbital edema after first dose	1	IM Adrenaline x two doses, IV hydrocortisone, IV diphenhydramine, nebulised salbutamol
2	39	F	Chinese	30 min	First	Asthma, allergic rhinitis	Flushing, erythema, breathlessness, globus sensation, wheezing * Had triphasic reaction with symptoms recurring 8 and 27 h post vaccination	1	IM adrenaline, IV hydrocortisone, IV diphenhydramine, IV cimetidine, nebulised salbutamol and ipratropium
3	42	F	Chinese	20 min	Second	Chronic rhinosinusitis	Generalised urticaria, periorbital edema, globus sensation, breathlessness	2	IM adrenaline, IV hydrocortisone and diphenhydramine
4	80	F	Chinese	30 min	First	Nil	Flushing, erythema, globus sensation, breathlessness, vomiting	2	IV hydrocortisone and diphenhydramine
5	26	F	Chinese	45 min	First	Allergic rhinitis, shellfish allergy	Urticaria, angioedema, breathlessness, giddiness, vomiting	2	IM adrenaline, IV hydrocortisone, PO chlorphenamine
6	52	F	Chinese	25 min	First	NSAID hypersensitivity, asthma, allergic rhinitis, peanut allergy	Periorbital edema, globus sensation, wheezing, hypotension	1	IM adrenaline x two doses, promethazine, Nebulised salbutamol
7	35	F	Indian	20 min	First	NSAID hypersensitivity, allergic rhinitis, episodic contact urticaria	Urticaria, globus sensation, breathlessness, hypotension	1	IV hydrocortisone, cimetidine, diphenhydramine, fluids
8	39	F	Chinese	10 min	First	Allergic rhinitis, chronic spontaneous urticaria, eczema, shellfish allergy	Epiglottic swelling, globus sensation, breathlessness, abdominal discomfort and diarrhoea	2	IM adrenaline x two doses, IV dexamethasone, diphenhydramine
9	38	F	Chinese	30 min	First	Asthma, allergic rhinitis, NSAID hypersensitivity	Urticaria, globus sensation, shortness of breath	1	Unknown (received initial treatment outside the institution)
10	38	M	Chinese	6 min	Second	Asthma	Urticaria, breathlessness, wheezing * Had mild breathlessness and wheezing after dose 1	1	IV diphenhydramine, hydrocortisone, salbutamol

Abbreviations: N.A., Not Available; IM, Intramuscular; IV, Intravenous; PO, Oral; NSAID, Non-steroidal anti-inflammatory drugs. * Additional signs and symptoms unique to the patient.

**Table 3:** (**a**) Immunological findings in vaccine non-reactors and vaccine reactors. (**b**) Immunological findings in vaccine non-reactors and six anaphylaxis patients with samples taken within 6 h of the hypersensitivity reaction.

(a)					
	Vaccine Non-Reactors *N* = 67	Vaccine Reactors Combined *N* = 47	*p* Value (Comparing Vaccine Non-Reactors and Vaccine Reactors Combined)	* Vaccine Non-Reactors 1 h after 1^st^ Dose BNT162b2 Vaccine *N* = 31	* Vaccine Non-Reactors 1 h after 2nd Dose BNT162b2 Vaccine *N* = 36
Anti-BNT162b2 IgG (ng/mL)	230.6 (77.6–449)	222.0 (118–360)	0.897	202.9 (75.7–377.5)	249.7 (95.8–482.7)
Anti-BNT162b2 IgM (AU/mL)	23.9 (13.8–48.9)	67.2 (36.2–91.6)	<0.001	20.9 (14.6–35.6)	34.8 (15.3–52.7)
Anti-BNT162b2 IgE (ng/mL)	N.D.	N.D.	N.A.	N.D.	N.D.
C5a (ng/mL)	39.4 (27.5–50.7)	160.0 (42.2–728.0)	<0.001	36.1 (24.5–45.0)	43.7 (32.6–52.1)
ICAM-1 (ng/mL)	46.0 (39.5–66.2)	136.0 (99.9–170.0)	<0.001	42.6 (37.1–63.4)	44.0 (39.5–56.3)
(**b**)					
	**Vaccine Non-Reactors** ***N*** **= 67**	**Anaphylaxis** **Patients** ***N*** **= 6**	***p*** **Value**		
Anti-BNT162b2 IgG (ng/mL)	230.6 (77.6–449)	372.6 (298.6–1169.8)	<0.05		
Anti-BNT162b2 IgM (AU/mL)	23.9 (13.8–48.9)	50.0 (30.3–56.9)	<0.05		
Anti-BNT162b2 IgE (ng/mL)	N.D.	N.D.	N.A.		
C5a (ng/mL)	39.4 (27.5–50.7)	571.6 (448.6–625.1)	<0.05		
ICAM-1 (ng/mL)	46.0 (39.5–66.2)	126.0 (89.0–135.0)	<0.05		

Data are expressed as median (IQR), IgE detection limit <0.2 ng/mL. Values used in vaccine non-reactors were samples taken 1 h after first or second dose of BNT162b2 vaccine. * Subcategories of vaccine non-reactors after first or second dose vaccination are presented. We find that there was no significant difference between these two groups. Unvaccinated subjects (n = 24), taken in 2019 prior to COVID-19 pandemic: Anti-BNT162b2 IgG 105.9 ng/mL (65.0–217.8) median and 95% CI; Anti-BNT162b2 IgM 17.9 AU/mL (11.7–28.4). Data are expressed as median (IQR), IgE was below detection limit < 0.2 ng/mL, non-detectable (N.D.).

**Table 4:** Comparison of vaccine reactors with high C5a vs. low C5a.

	Vaccine Reactors with High C5a (*N* = 37)	Vaccine Reactors with Low C5a (*N* = 8)	*p* Value
C5a (ng/mL) *	697 (271–1147)	37.6 (27.0–49.1)	<0.001
Anti-BNT162b2 IgG (ng/mL)	302 (200–417)	292 (148–491)	0.999
Anti-BNT162b2 IgM (AU/mL)	62.8 (30.7–80)	50.3 (28–75.5)	0.961
Anti-BNT162b2 IgE (ng/mL)	N.D.	N.D.	N.A.
ICAM-1 (ng/mL)	119 (68.6–146)	105 (68.9–141)	0.999
IL-4 (pg/mL)	5.65 (1.21–46.0)	0.29 (0.15–2.33)	<0.02
IL-10 (pg/mL)	9.52 (8.97–15.6)	2.69 (2.41–6.44)	0.456
IL-33 (pg/mL)	9.34 (4.4–86.4)	2.34 (0.52–5.05)	<0.003
TNF (pg/mL)	0.54 (0.39–1.1)	7.02 (2.88–19.9)	<0.042
MCP-1 (pg/mL)	308 (252–412)	31.6 (16.2–233)	<0.003

* C5a levels from the 67 vaccine non-reactors, where 95% of the data fell within mean + 2.5 standard derivations, with the final cut-off of 76.6 ng/mL defined as high C5a. Data are expressed as median (IQR), normal range: IL-4, IL-10, IL-33 and TNF (<2 pg/mL), ICAM-1 (<95 ng/mL), MCP-1 (163 pg/mL, 134–197). IgE detection limit <0.2 ng/mL.

**Table 5:** Basophil activation test (BAT) in five anaphylaxis patients.

Patient No	Experiments	CCR^+^/CD63^+^ Basophil (%)	CCR3^+^/CD 203c^+^ Basophil (%)
Patient 1 *	Negative control (buffer solution)	0.5	0.9
	Anti-IgE positive control	7.8 *	20.3
	Pfizer-BNT162b2 1%	0.1	1.4
	Pfizer-BNT162b2 0.1%	0.7	1.7
Patient 2	Negative control (buffer solution)	0.2	0.4
	Anti-IgE positive control	81.7	56.2
	Pfizer-BNT162b2 1%	0.5	0.9
	Pfizer-BNT162b2 0.1%	0.2	0.8
Patient 3	Negative control (buffer solution)	0.2	0.5
	Anti-IgE positive control	84.6	49.5
	Pfizer-BNT162b2 1%	0	1.5
	Pfizer-BNT162b2 0.1%	0.1	0.7
	PEG 2000 1%	0.2	0.1
	DMG-PEG 2000 1%	0.3	0.4
Patient 4	Negative control (buffer solution)	0.1	0.2
	Anti-IgE positive control	69.2	65.2
	Pfizer-BNT162b2 1%	0.2	0.6
	Pfizer-BNT162b2 0.1%	0.1	0.4
	PEG 2000 1%	0.2	0.1
	DMG-PEG 2000 1%	0.6	0.4
Patient 5	Negative control (buffer solution)	0.1	1.2
	Anti-IgE positive control	50.4	39.9
	Pfizer-BNT162b2 1%	0.2	0.7
	Pfizer-BNT162b2 0.1%	0.1	0.5
	PEG 2000 1%	0.2	0.7
	DMG-PEG 2000 1%	0.1	0.4

* According to the criteria set by Bülhmann Laboratories AG, the samples are evaluable if the positive control stimulator (anti-FceR1 mAb) shows activation of >10% for CCR^+^/CD63^+^ and >5% for CCR^+^/CD203c^+^ basophils, respectively. Out of the five samples performed, four (patients 2 to 5) fulfilled this requirement. Patient 3 to 5 basophils were stimulated with PEG-2000 (1%) and DMG PEG-2000 (1%) in addition to the Pfizer BNT162b2 vaccine at 0.1% and 1%, respectively. A positive BAT is defined as an absolute activated basophil percentage of >5%.

## Data Availability

The data presented in this study are available on request from the corresponding author. The data are not publicly available due to privacy and ethical reasons.

## Supplementary Materials

The following supporting information can be downloaded at: https://www.mdpi.com/article/10.3390/vaccines11061020/s1, Table S1: Laboratory findings of 10 patients with anaphylaxis in response to Pfizer BNT162b2 vaccine.
