# Supplementary material for: Anaphylatoxin Complement 5a in Pfizer BNT162b2-Induced Immediate-Type Vaccine Hypersensitivity Reactions
Source: Vaccines (Basel). 2023 May 23;11(6):1020. doi: 10.3390/vaccines11061020 (PMC10301384; doi:10.3390/vaccines11061020)
Supplement: Supplementary file 1 [file vaccines-11-01020-s001.zip › vaccines-2360499-supplementary.pdf]

Supplementary appendix

Table S1. Laboratory findings of 10 patients with anaphylaxis to Pfizer BNT162b2 vaccine

S/N	**Time of blood collection after the onset of anaphylaxis**	**Tryptase Ng/ml**	**C5a Ng/ml**	**ICAM-1 Ng/ml**	**Anti-BNT162b2 IgG ng/ml**	**Anti-BNT162b2 IgM (AU/ml)**	**Anti-BNT162b2 IgE (ng/ml)**	**WBC 10^9/L**	**Lymphocytes 10^9/L**	**Hb g/dL**	**Plt 10^9/L**
1	3 hours	3.2	448.6	138.0	83.0	34.4	N.D.	10.6	0.93	15.9	414
2	6 hours	2.2	571.6	56.5	290.8	59.1	N.D.	10.5	3.89	13.9	380
3	6 hours	2.3	696.8	164.0	2348.0	182.5	N.D.	8.2	3.08	14.0	413
4	5 hours	5.1	625.1	126.0	1436.0	25.2	N.D.	3,9	0.91	11.8	221
5	48 hours	1.8	40.8	80.3	1144.0	21.7	N.D.	7.5	2.35	13.4	315
6	5 hours	3.4	49.1	86.3	322.0	29.0	N.D.	13.9	5.04	13.0	517
7	72 hours	4.4	49.1	69.4	316.2	63.4	N.D.	N.A.	N.A.	N.A.	N.A.
8	5 hours	6.3	572.5	97.2	372.6	53.4	N.D.	8.4	3.42	13.1	243
9	42 hours	3.9	21.0	98.0	288.8	50.0	N.D.	6.8	0.91	13.6	282
10	70 hours	2.9	50.0	106.7	251.6	29.9	N.D.	N.A.	N.A.	N.A.	N.A.

Baseline tryptase was followed up subsequently and the acute tryptase samples did not show significant rise (> 1.2x baseline tryptase + 2 ng/L)

Abbreviations: N.A., Not Available; N.D., Not Detectable; WBC, White Blood Cell; Hb, Haemoglobin; Plt, Platele
